# The relationship between parent-child relationship and prosocial behavior among Chinese children: the mediating roles of labor participation and sense of social responsibility

**DOI:** 10.3389/fpsyg.2026.1804412

**Published:** 2026-07-13

**Authors:** Xurui Zhang, Weiwei Tang, Qingqing Miao, Haitian Lan, Fangqi Wang, Shiqi Wang, Jing Tao, Shuiping Deng, Rui Wang, Jinhua Gu, Cheng Chen, Zinan Zhang, Xin Cao

**Affiliations:** 1 School of Public Health, Nantong University, Nantong, Jiangsu, China; 2 School of Health Policy and Management, Nanjing Medical University, Nanjing, Jiangsu, China; 3 Nantong First People’s Hospital, Nantong, China; 4 Department of Infectious Disease Prevention and Control, Pingliang Kongtong District Center for Disease Control and Prevention, Pingliang, Gansu, China; 5 The Affiliated Taizhou People’s Hospital of Nanjing Medical University, Taizhou, Jiangsu, China; 6 Taizhou School of Clinical Medicine, Nanjing Medical University, Taizhou, Jiangsu, China; 7 Graudate School of Nanjing Medical University, Nanjing, Jiangsu, China

**Keywords:** Chinese children, labor participation, parent-child relationship, prosocial behavior, sense of social responsibility

## Abstract

**Purpose:**

Based on ecosystem theory, this study explores the relationship between parent-child relationships and prosocial behavior among primary students, as well as the mediating effect of labor participation and sense of social responsibility.

**Methods:**

A total of 810 primary students completed a self-report questionnaire including the Chinese Parent Intimacy Questionnaire-child (PIQ-C), Labor Participation Questionnaire (LPQ), Social Responsibility Status Questionnaire (SRSQ), and Chinese Prosocial Tendencies Measure (PTM-C). For mediation exploration, a serial mediation model was utilized, and the Bootstrap method was employed to test the significance of these mediation effects.

**Results:**

Parent-child relationship demonstrates a positive correlation with prosocial behavior. Through the father-child relationship, three significant mediation pathways were identified that directly affect prosocial behavior: (1) labor participation (2) sense of social responsibility (3) labor participation and sense of social responsibility. Research finds that father-child relationships tend to directly influence children’s behavioral patterns, while mother-child relationships may have a more structural effect on children’s social-emotional functioning.

**Conclusion:**

Labor participation and sense of social responsibility were identified as serial mediators in the relationship between parent-child relationships and prosocial behaviors.

## Introduction

Prosocial behavior refers to the behavior that conforms to social norms and has a positive impact on people and society, including assistance, sharing, cooperation, donations, etc., (Eisenberg et al., 2007). Children’s prosocial behavior serves as a conspicuous manifestation of the maturation of personality traits and moral values, as well as a pivotal indicator of social development (Tintori et al., 2021). Children who frequently exhibit prosocial behaviors are more likely to experience more positive emotions and fewer negative emotions, (Snippe et al., 2018) have a stronger sense of well-being, (Weinstein and Ryan, 2010) and have better physiological functions and health (Brown and Brown, 2015; González Moreno and Molero Jurado, 2024). They are better able to withstand the effects of stressful events (Raposa et al., 2016) and have higher quality interpersonal and intimate relationships (Son and Padilla-Walker, 2020). They will be more easily recognized and accepted by others, which will effectively reduce the occurrence of problem behaviors such as social withdrawal and aggression, and help children better adapt to society (Miconi et al., 2019). Middle and late childhood is the key stage to cultivate children’s prosocial behavior. Studies have shown that children aged 6–12 are generally able to display prosocial behavior, (Salerni and Caprin, 2022) while children aged 8–12 show a higher frequency of prosocial behavior (Ferschmann et al., 2024).

Prior studies have emphasized various factors that contribute to the formation of prosocial importance in behavior. These factors include personality traits, (Graziano et al., 2007) parental affection (Knafo and Plomin, 2006) and social influence (Liu et al., 2021). In addition, studies have demonstrated a strong association between parent-child attachment and prosocial behavior, (Gross et al., 2017; Mikulincer and Shaver, 2007) with a positive relationship being a strong indicator of children’s prosocial behavior (Ferreira et al., 2016). Enhanced parent-child closeness can positively impact the development of children’s prosocial behavior by bolstering their social competence (Laursen and Collins, 2009).

Ecological system theory (Bronfenbrenner, 1979) emphasizes that the interaction between individual characteristics and ecological systems (e.g., family and society) shapes the developmental outcomes of individuals. However, current research is unclear in terms of causality and psycho-behavioral mechanisms (Costa Martins et al., 2022). It is necessary to underlying mechanisms of action and influences related to children’s pro-social behavior. This study aimed to examine the influence of parent-child relationships, labor participation, and sense of social responsibility on children’s prosocial behavior, relying on the principles of ecosystem theory. It will offer insights for future treatments focused on improving children’s prosocial behaviors and for the advancement of education in primary schools.

## Literature review and hypothesis development

### Parent-child relationship in relation to prosocial behavior

The parent-child relationship is a fundamental interpersonal connection that emerges through interactions within families, influenced by biological and genetic factors. As the primary social connections individuals experience, parent-child relationships play a crucial role in shaping various aspects of an individual’s personality development, social cognition, and mental well-being (Maccoby, 1992). Previous research suggests that a robust parent-child relationship is essential for promoting a healthy childhood and serves as a key protective factor for overall development (Barber et al., 2005). According to attachment theory, (Barbour, 1969) early experiences of attachment to others have an impact on their thought and behavior patterns in later relationships (Costa Martins et al., 2022). Previous studies have shown that prosocial behavior in children is closely correlated with the degree of intimacy of family members (Kerns et al., 2000) and that prosocial behavior and parent-child relationships are strongly correlated (Nie et al., 2016).

Parents have varying effects on the development of their children because of the division of labor within the family and the social responsibilities they take on. Elhusseini et al. (2023) found that father-child attachment security was associated with greater prosocial behavior in middle childhood, and that child emotional dysregulation served as an intervening variable in this association. Specifically, more secure father-child attachment was related to lower emotional dysregulation, which in turn was associated with higher prosocial behavior (Elhusseini et al., 2023). Mothers’ negative interactions and disconfirming behaviors were found to be negatively correlated with prosociality (Piotrowski, 2024). Therefore, we treated the mother-child and father-child relationships as two distinct dimensions for measurement and observation. Based on the above, the following hypotheses are proposed:

**H1a** Father-child relationship has a positive predictive effect on prosocial behavior.

**H1b** Mother-child relationship has a positive predictive effect on prosocial behavior.

### Mediation effect of labor participation

Labor is a distinct human endeavor that enables individuals to modify their interactions with the natural world. Compared to the Industrial Revolutionary period, modern society has seen a dramatic shift in the way children live: children leave the productive sector and enter schools, where the main duty of children is to learn and to enjoy their childhood (Riadigos and Gradaille, 2023). Children’s right to education has been greatly guaranteed. Still, they have consequently had fewer opportunities to freely access and explore the natural world and the surrounding community, and have lost the experience of real labor and socialization. Accordingly, the labor proposed in this paper refers specifically to labor in an educational context. It refers to children’s active engagement in age-appropriate productive activities, encompassing self-service labor, family service labor, and service work inside or outside the school. We quantitatively assess specific types or categories of activities in children’s labor participation, including the extent, frequency, and duration of participation (Fredricks et al., 2014).

According to ecosystem theory, the family as a microsystem of personal development has a direct influence on children. Several studies have revealed that labor consciousness is initially cultivated in the family, (Wang et al., 2022) and household chores and parent-child relationships are strongly correlated (Li S., 2016). Research on the consequences of housework revealed that it can offer children chances for self-directed learning and cultivate a sense of responsibility (Goodnow, 1988; Grusec, 2011). Sociologists point out that children’s involvement in household chores is a crucial aspect of family socialization as well as a facilitator of prosocial behavior (Gu and Chao, 2022; Lareau and Weininger, 2003; Rheingold, 1982). In China, the situation of children’s labor participation exhibits a greater diversity. Some researchers highlight that labor education should include production labor education, service labor education, and daily life labor education (Liu, 2019). Therefore, labor participation mentioned in this study is defined as an indicator of an individual’s involvement in any form of labor activity. Based on the above, the following hypotheses are proposed:

**H2a** Labor participation mediates the relationship between the father-child relationship and prosocial behavior.

**H2b** Labor participation mediates the relationship between the mother-child relationship and prosocial behavior.

### Mediation effect of sense of social responsibility

Sense of social responsibility is conceptualized as a set of values and personal commitments that entail assisting or caring for others, including strangers, and actively contributing to society to enhance one’s community and the larger society (Bartolo et al., 2023). The concept of the sense of social responsibility has received significant attention in the field of psychology due to its positive impact on subject well-being, (Wu et al., 2022) social and academic performance, (Carbonero et al., 2017) and prosocial behavior (O’connor and Cuevas, 1982). Eisenberg’s prosocial moral theory (Snippe et al., 2018) posits that prosocial moral reasoning progresses from hedonistic reasoning to internalized moral principles, with moral emotion as a fundamental determinant of children’s prosocial behavior. Sense of social responsibility, as examined in this study, corresponds to the internalized stage in which individuals act prosocially based on a sense of duty rather than external rewards. Massive evidence indicates that a sense of social responsibility in adolescence is closely related to prosocial behavior (Bartolo et al., 2023; Silke et al., 2021). Current research indicates that a greater sense of social responsibility predicts more prosocial behavior and improves an individual’s mental health (Jiang et al., 2021).

Furthermore, family, as the primary nurturing environment for children, plays a crucial role in enhancing pupils’ sense of personal responsibility (Cheng et al., 2021). The quality of the parent-child relationship directly affects children’s future behavior patterns and development trajectories (Zhu et al., 2023). A previous study found that a good parent-child relationship promotes children’s active participation in family life and greater social responsibility (Koljatic et al., 2021). Positive interaction, deep affection, mutual understanding, and trust between parents and children also help to cultivate children’s sense of social responsibility (Maiya et al., 2023; Zhu et al., 2023). Rooted in Confucian values and collectivistic orientations, Chinese society places strong emphasis on individuals’ obligations to family, community, and the broader social collective (Cheng et al., 2021). Children in China are socialized from an early age to prioritize group harmony and collective well-being, and concepts such as filial piety and reciprocal obligation are woven into everyday family interactions and educational practices. This cultural backdrop suggests that the development of social responsibility among Chinese children may be closely tied to family socialization processes.

Middle childhood is a particularly relevant developmental period for examining these pathways. During this stage, children develop advances in perspective-taking and moral reasoning that enable them to internalize social values rather than merely complying with external rules (Eisenberg et al., 2007). Meanwhile, their social contexts expand beyond the family into school and peer settings, making this a critical window for understanding how family relationships shape the internalization of social responsibility. Taken together, both the cultural emphasis on collective obligation and the cognitive-developmental characteristics of middle childhood suggest that sense of social responsibility may serve as a key mechanism linking parent-child relationships to prosocial behavior. Based on the above, the following hypotheses are proposed:

**H3a** Sense of social responsibility mediates the relationship between the father-child relationship and prosocial behavior.

**H3b** Sense of social responsibility mediates the relationship between the mother-child relationship and prosocial behavior.

### Chain mediating role of labor participation and sense of social responsibility

By specifically examining the mediating role of labor participation and sense of social responsibility between the parent-child relationship and prosocial behavior, people may inadvertently overlook the intrinsic connection between children’s labor participation and social responsibility.

Labor participation serves as a predictive index for social responsibility. Previous research indicates that the sharing of household chores not only significantly alleviates the burden on parents but also fosters essential life skills and promotes physical health among children (Li S. A, 2016). Importantly, this process further strengthens their sense of responsibility and belonging to both family and society (Hossain et al., 2023; White and Brinkerhoff, 1981). Children who infrequently engage in household chores were more likely to score in the lowest quintile on self-reported prosocial behavior, academic ability, peer relationships, and life satisfaction compared to their peers who regularly performed chores (White et al., 2019). It is worth noting that by performing basic duties, these youngsters gained a sense of competence, which makes them remain committed to undertaking housework (Wang et al., 2022). A survey of adolescents’ participation in extracurricular activities also shows that adolescents’ participation in extracurricular activities is related to their psychological and behavioral functions (Bartko and Eccles, 2003). These studies reveal that children’s involvement in household chores and extracurricular activities is significantly linked to improved academic outcomes, psychosocial functioning, and prosocial behaviors. Furthermore, chores play a crucial role in cultivating children’s grateful hearts, which is also related to a good parent-child relationship (Li S., 2016). To sum up, it implies that the labor participation and social responsibility meditation models are not independent. Consequently, we hypothesized that parent-child relationship promotes labor participation among children, which, in turn, leads to stronger social responsibility and ultimately a higher level of prosocial behavior. Based on the above, the following hypotheses are proposed:

**H4a** Father-child relationship can positively predict prosocial behavior through the chain mediating effect of labor participation and sense of social responsibility.

**H4b** Mother-child relationship can positively predict prosocial behavior through the chain mediating effect of labor participation and sense of social responsibility.

**FIGURE 1:**
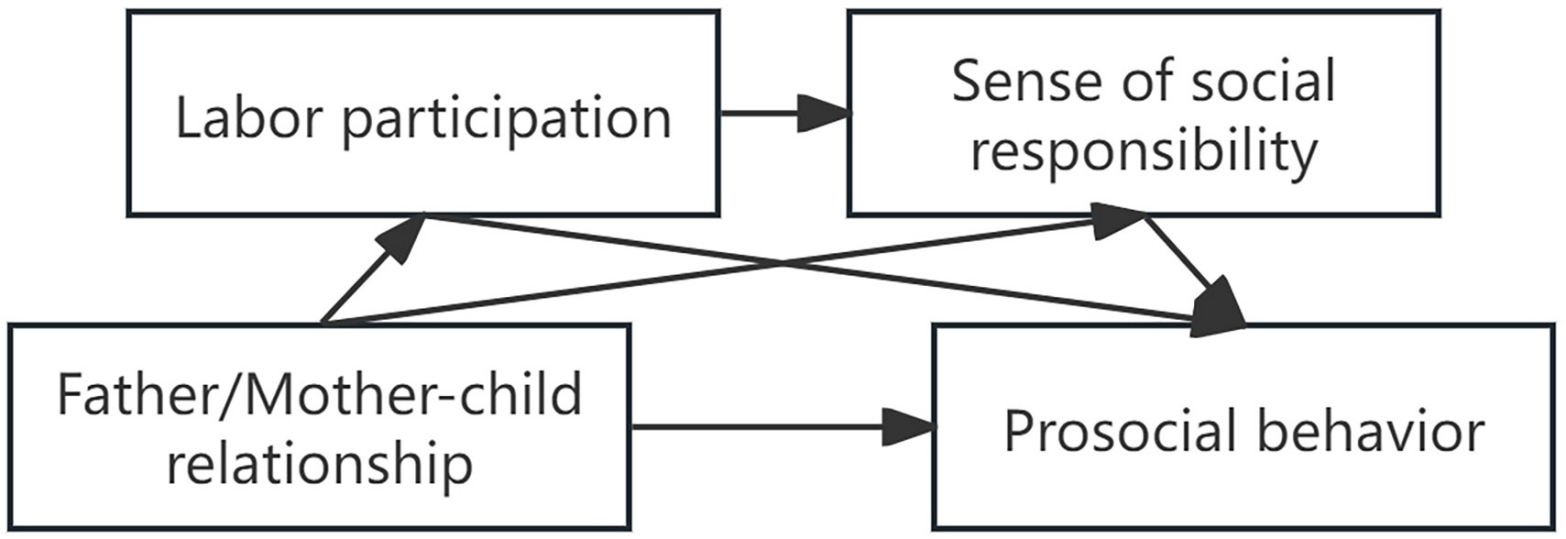
Hypothesized moderated mediation model.

According to the literature and theory mentioned above, parent-child relationship, labor participation, social responsibility and prosocial behavior are pairwise related, but there are still unclear results for the possible mechanisms underlying these associations among Chinese children. Therefore, a chain mediation model (see Figure 1) was proposed to test the relationship between the father-child relationship/mother-child relationship and prosocial behavior.

## Methodology

### Participants and procedure

Multi-stage stratified cluster sampling method was conducted as follows: first, the present study randomly opted for students from 2 primary schools in Jiangsu and Gansu provinces from December 2023 to January 2024; second, due to the limited ability of 1st and 2nd-grade students to independently complete questionnaires, the random number method was used to select 18 classes to investigate the students in grades 3–6; lastly, at least 80 students were randomly selected in each grade. The whole survey lasted approximately 30 min. After the participants completed the questionnaire, the questionnaire was collected and checked by the investigator.

A total of 810 questionnaires were collected, of which 805 cases (99.38%) were valid. In the current sample (*N* = 805), the average age of participants was 10.07 ± 1.26, 390 (48.45%) were girls and 415 (51.55%) were boys. Regarding the areas of participants, 428 (53.17%) participants were in the eastern region, and 377 (46.83%) participants were in the western region.

### Ethical approval and informed consent

This project was approved by the ethics committee of Nantong University (Approval No. 202444). All participants, including school administrators, teachers, and students, provided informed consent prior to the interview surveys and data collection. Given the complexity of collecting data, these instructions and procedures were introduced by trained investigators before the survey. In addition, we ensured the anonymity and confidentiality of the information collected. Participation in the study was entirely voluntary, and participants were informed of their right to withdraw at any time without providing an explanation or facing any negative consequences.

### Measurement

#### Parent-child relationship: Parent Intimacy Questionnaire-Child (PIQ-C)

Parent-child relationship was measured using the Chinese version of “Parent Intimacy Questionnaire-child” (PIQ-C) (Buchanan et al., 1991; Xu et al., 2018). It is an 18-item scale and is divided into mother-child relationship and father-child relationship, with each containing 9 items and two contents consistent. Children rated each item separately for their father and mother, providing distinct scores for each parent. This design acknowledges that children may perceive different levels of closeness with each parent. Each item was presented twice, once referring to the father and once to the mother, so that children could provide independent ratings for each parental relationship. Children rated items on a 5-point Likert scale ranging from 1 (never) to 5 (always), prompted to evaluate the frequency of specific interactions with their father/mother. Example items from the father-child subscale include *“Do you feel close to your dad?” (trust)*, *“My father helps me to talk about my difficulties” (communication)*. Parallel items on the mother-child subscale ask the same questions with reference to the mother. The total score of each subscale is calculated independently, with higher scores reflecting higher relationship quality with that specific parent. The PIQ-C was used to indicate parent-child relationship quality in the current study. Alpha for the PIQ-C in the current study was good (α = 0.920, *p* < 0.001), and the internal consistency coefficients of the two dimensions were 0.892 (father-child relationship) and 0.870 (mother-child relationship), respectively.

#### Prosocial behavior: Prosocial Tendencies Measure (PTM-C)

Prosocial behavior was measured using the Chinese version of the Prosocial Tendencies Measure (PTM-C) (Kou et al., 2007). It is a 26-item scale derived from the Prosocial Tendencies Measure, (Carlo and Randall, 2002) consisting of 6 forms of prosocial behavior: dire, emotional, public, altruistic, anonymous and compliant. Children indicated how well each statement described their helping tendencies on a 5-point scale from 1 (*strongly disagree*) to 5 (*strongly agree*). Example items: *“I tend to help people who are in a real crisis or need” (dire), “I respond best when the situation is highly emotional” (emotional), “When people ask me to help, I don’t hesitate” (compliant), “I can help others best when people are watching me”(public), “I tend to help others when they do not know who helped them” (anonymous), “I often help others even when there is no benefit to me” (altruistic)*. The scale with a total score of 130 points was used to indicate their likelihood of becoming involved in prosocial behavior in the current study. Higher scores indicate higher prosocial tendencies. The PTM-C was used to assess prosocial behavior among primary school students. Alpha for the PTM-C in the current study was good (α = 0.943, *p* < 0.001).

#### Labor participation: Labor Participation Questionnaire (LPQ)

Labor participation was evaluated by “Labor Participation Questionnaire” (LPQ), which was compiled by Pang (2022) according to the situation of primary school students in China (LPQ). It is a 21-item self-report measure that assesses three dimensions of labor participation among primary school students: self-service labor, family service labor and service work inside or outside the school.

Children reported how often they participated in various activities over the past year. Items are rated on a 4-point scale ranging from 1 (*almost nothing*) to 4 (*almost every day*). Example items include *“Organizing your room” (self-service)*, “Washing the dishes” (family service) *“Participation in school radio stations, blackboards, handbills and other publicity activities” (service work inside or outside the school)*. The number of times students participate in labor activities periodically represents the frequency of labor participation. The total score of the questionnaire is 84 and higher scores reflect higher levels of labor participation. The LPQ was used to assess labor participation among primary school students. Alpha for the LPQ in the current study was good (α = 0.902, *p* < 0.001).

#### Sense of social responsibility: Social Responsibility Status Questionnaire (SRSQ)

Sense of Social responsibility was measured by the “Social Responsibility Status Questionnaire” (SRSQ) (Hu, 2022). It is a 30-item self-report measure that assesses five dimensions of social responsibility in primary school students: personal responsibility (1–6), family responsibility (7–12), others’ responsibility (13–18), collective responsibility (19–24) and social and public responsibility (25–30). Items are rated on a 5-point scale ranging from 1 (*strongly disagree*) to 5 (*strongly agree*). Children were asked to rate how much they agree with each statement based on their actual behavior. Example items include *“I set learning goals for myself” (personal responsibility), “I actively do things within my ability for my family” (family responsibility), “I am willing to help classmates who have difficulties” (others’ responsibility), “I think the collective honor is related to each student” (collective responsibility)*, and *“I actively participate in public welfare activities” (social and public responsibility)*. The total score of the questionnaire is 150 points, with each dimension 30 points. The higher the score, the better the students are in a certain aspect of social responsibility. The SRSQ was used to assess social responsibility among primary school students. Alpha for the SRSQ in the current study was good (α = 0.944, *p* < 0.001).

### Data analysis

Stata software (v.17.0) and IBM SPSS Statistics (v.26.0) were used for data analysis. First, descriptive statistics and Pearson correlations between the variables of interest were initially calculated. Next, the mediating role of labor participation and social responsibility on the effect of the father/mother-child relationship on prosocial behavior was assessed using SPSS macro PROCESS (Model 6) v4.1 by Hayes (2013). In addition, the direct and indirect effects were tested for statistical significance by generating percentile bootstrap confidence intervals (95% CI) based on 5000 resamples from the data, with the effects being considered significant if the CIs did not include zero. The significance level of the current study was set as 0.05.

The data collection method employed in this study involved self-reports, which have the potential to introduce systematic errors. To ensure the scientific rigor of the study’s findings, Harman’s single-factor test was conducted to assess the presence of common method bias before the formal data analysis (Morrison and Harman, 1961). The results of Harman’s single-factor test showed that the exploratory factor analysis extracted 17 factors (Eigenvalues > 1), and the largest factor explained 25.16% of the variance, which complied with the critical condition of less than 40%, thus indicating the absence of significant common method bias in the study.

## Results

### Descriptive and correlational analyses

The results of means, standard deviations, and correlation of each variable are given in Table 1. In this study, the mean score for the father-child relationship was 31.49 ± 9.51. The mean score for mother-child relationship was 34.17 ± 8.41, and mother-child relationships scored higher than father-child relationships. The mean score for labor participation was 57.54 ± 12.68. The mean score for the sense of social responsibility was 134.61 ± 15.94. The mean score for prosocial behavior was 109.16 ± 17.43. According to these findings, it was determined that there were significant positive correlations for father-child relationship with mother-child relationship (*r* = 0.66, *p* < 0.01), labor participation (*r* = 0.35, *p* < 0.01), sense of social responsibility (*r* = 0.36, *p* < 0.01) and prosocial behavior (*r* = 0.33, *p* < 0.01); for mother-child relationship with labor participation (*r* = 0.30, *p* < 0.01), sense of social responsibility (*r* = 0.39, *p* < 0.01) and prosocial behavior (*r* = 0.32, *p* < 0.01); for labor participation with social responsibility (*r* = 0.52, *p* < 0.01) and prosocial behavior (*r* = 0.45, *p* < 0.01); and finally, for sense of social responsibility with prosocial behavior (*r* = 0.70, *p* < 0.01).

**TABLE 1:** Descriptive statistics and inter-correlations for the study variables (*N* = 805).

Variables	1	2	3	4	5	M ± SD
1. Father-child relationship	1	1	1	1	1	31.49 ± 9.51
2. Mother-child relationship	0.66**					34.17 ± 8.41
3. Labor participation	0.35**	0.30**				57.54 ± 12.68
4. Sense of social responsibility	0.36**	0.39**	0.52**			134.61 ± 15.94
5. Prosocial behavior	0.33**	0.32**	0.45**	0.70**		109.16 ± 17.43

**p < 0.01.

### Serial mediation model results

**FIGURE 2:**
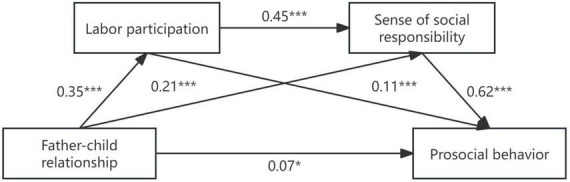
Father-child relationship and prosocial behavior: a serial mediation model. ***p < 0.001; *p < 0.05.

**FIGURE 3:**
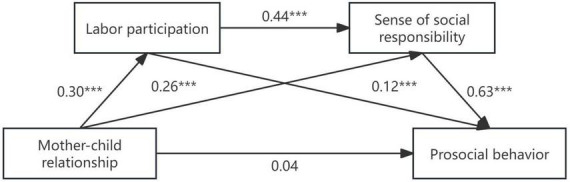
Mother-child relationship and prosocial behavior: a serial mediation model. ***p < 0.001.

The results of the serial mediation model are illustrated in Figures 2, 3 and Table 2. Father-child relationship was positively associated with prosocial behavior in the absence of mediating variables (β = 0.33, *p* < 0.001), thus supporting H1a. The results of the mediating effect analysis (see Figure 2 and Table 2) showed that father-child relationship was positively associated with labor participation (β = 0.35, *p* < 0.001), sense of social responsibility (β = 0.21, *p* < 0.001) and prosocial behavior (β = 0.07, *p* < 0.05). Labor participation was positively associated with sense of social responsibility (β = 0.45, *p* < 0.001) and prosocial behavior (β = 0.11, *p* < 0.001). In addition, a sense of social responsibility was also positively associated with prosocial behavior (β = 0.62, *p* < 0.001). These results indicated that the serial mediating effect of “labor participation → sense of social responsibility” was significant among the influences of father-child relationship on prosocial behavior, thus supporting H2a, H3a and H4a.

**TABLE 2:** Regression analysis of the relationship between variables in two chains mediation model.

Result variables	Predictive variables	R	R^2^	F	β	SE	t	95% CI
Prosocial behavior	Father-child relationship	0.33	0.11	95.90***	0.33	0.06	9.79***	[0.48, 0.72]
Labor participation	Father-child relationship	0.35	0.12	108.39***	0.35	0.04	10.41***	[0.37, 0.55]
Sense of social responsibility	Father-child relationship	0.55	0.31	175.90***	0.21	0.05	6.55***	[0.24, 0.45]
	Labor participation				0.45	0.04	14.24***	[0.48, 0.64]
Prosocial behavior	Father-child relationship	0.71	0.51	272.95***	0.07	0.05	2.45*	[0.02, 0.22]
	Labor participation				0.11	0.04	3.57***	[0.07, 0.23]
	Sense of social responsibility				0.62	0.03	20.88***	[0.62, 0.74]
Prosocial behavior	Mother-child relationship	0.32	0.10	89.11***	0.32	0.07	9.44***	[0.52, 0.79]
Labor participation	Mother-child relationship	0.30	0.09	77.38***	0.30	0.05	8.80***	[0.35, 0.55]
Sense of social responsibility	Mother-child relationship	0.57	0.33	196.79***	0.26	0.06	8.57***	[0.38, 0.61]
	Labor participation				0.44	0.04	14.55***	[0.48, 0.63]
Prosocial behavior	Mother-child relationship	0.71	0.50	270.20***	0.04	0.06	1.37	[−0.03, 0.19]
	Labor participation				0.12	0.04	3.94***	[0.08, 0.24]
	Sense of social responsibility				0.63	0.03	20.60***	[0.62, 0.75]

***p < 0.001;

*p < 0.05.

Similarly, the mother-child relationship quality significantly predicted higher levels of prosocial behavior in the absence of mediating variables (β = 0.32, *p* < 0.001), thus supporting H1b. The results of the mediating effect analysis (see Figure 3 and Table 2) showed that a better mother-child relationship significantly predicted better labor participation (β = 0.30, *p* < 0.001) and sense of social responsibility (β = 0.26, *p* < 0.001), but there was no significant effect on prosocial behavior (β = 0.04, *p* > 0.05). And better labor participation significantly predicted a higher sense of social responsibility (β = 0.44, *p* < 0.001) and prosocial behavior (β = 0.12, *p* < 0.001). Additionally, a higher sense of social responsibility significantly predicted higher prosocial behavior (β = 0.63, *p* < 0.001). These results indicated that the serial mediating effect of “labor participation → sense of social responsibility” was significant among the influences of mother-child relationship on prosocial behavior, thus supporting H2b, H3b and H4b.

### Bootstrap of mediators

As shown in Table 3, the results suggested that all three indirect paths were significant. The Father-child relationship had a significant total effect on prosocial behavior (effect = 0.60, 95% CI = [0.48, 0.72]). The total indirect effect was 0.48, accounting for 79.63% of the total effect. Specifically, the indirect effect contained three mediation pathways: (1) Path 1a: Father-child relationship→Labor participation→Prosocial behavior (effect = 0.07, 95% CI = [0.02, 0.11]), (2) Path 2a: Father-child relationship→Sense of social responsibility→Prosocial behavior (effect = 0.23, 95% CI = [0.16, 0.31]), (3) Path 3a: Father-child relationship→Labor participation→Sense of social responsibility→Prosocial behavior (effect = 0.18, 95% CI = [0.13, 0.22]). The three indirect effects accounted for 11.19%, 39.07%, and 29.38% of the total effect, respectively. After controlling for the two mediators, the direct effect of father-child relationship on prosocial behavior was still significant (effect = 0.12, 95% CI = [0.02, 0.22]), showing that there was a serial mediation model with two mediators in the relationship between father-child relationship and prosocial behavior. Based on the above results, the mediating variables were observed to partially mediate between the father-child relationship and prosocial behavior.

**TABLE 3:** Comparison of the indirect effects of father-child relationship on prosocial behavior through labor participation and sense of social responsibility.

Variable relationship	Effect	Boot SE	Boot LLCI	Boot ULCI	Mediated (%)
Total effect	0.60	0.06	0.48	0.72	–
Direct effect	0.12	0.05	0.02	0.22	–
Total indirect effect	0.48	0.05	0.38	0.58	79.63%
Path 1a: Father-child relationship→Labor participation→Prosocial behavior	0.07	0.02	0.02	0.11	11.19%
Path 2a: Father-child relationship→Sense of social responsibility→Prosocial behavior	0.23	0.04	0.16	0.31	39.07%
Path 3a: Father-child relationship→Labor participation→Sense of social responsibility→Prosocial behavior	0.18	0.02	0.13	0.22	29.38%

As seen from the results in Table 4, the mother-child relationship also had a significant total effect on prosocial behavior (effect = 0.66, 95% CI = [0.52, 0.79]). Labor participation and sense of social responsibility have significant mediating effects between mother-child relationship and prosocial behavior ((4) Path 1b: effect = 0.07, 95% CI = [0.03, 0.12]; (5) Path 2b: effect = 0.34, 95% CI = [0.25, 0.43]), accounting for 10.84% and 51.45% of the total effect, respectively. Meanwhile, labor participation and sense of social responsibility acted as chained mediation between mother-child relationship and prosocial behavior ((6) Path 3b: effect = 0.17, 95% CI = [0.12, 0.22]), accounting for 25.95% of the total effect. However, the direct effect of mother-child relationship on prosocial behavior was not statistically significant after controlling for the hypothesized mediators (effect = 0.08, 95% CI = [−0.03, 0.19]). According to these findings, labor participation and sense of social responsibility play a complete mediating role between the mother-child relationship and prosocial behavior.

**TABLE 4:** Comparison of the indirect effects of mother-child relationship on prosocial behavior through labor participation and social responsibility.

Variable relationship	Effect	Boot SE	Boot LLCI	Boot ULCI	Mediated (%)
Total effect	0.66	0.07	0.52	0.79	–
Direct effect	0.08	0.06	−0.03	0.19	–
Total indirect effect	0.58	0.06	0.47	0.69	88.09%
Path 1b: Mother-child relationship→Labor participation→Prosocial behavior	0.07	0.02	0.03	0.12	10.84%
Path 2b: Mother-child relationship→Sense of social responsibility→Prosocial behavior	0.34	0.05	0.25	0.43	51.45%
Path 3b: Mother-child relationship→Labor participation→Sense of social responsibility→Prosocial behavior	0.17	0.03	0.12	0.22	25.95%

## Discussion

This study employed a serial mediation model to examine the associations among parent-child relationships and prosocial behaviors in Chinese children. The findings of this study indicate that the prosocial behavior of Chinese children was significantly and positively associated with the parent-child relationship. Furthermore, participation in labor and a sense of social responsibility play a vital role in mediating the association between parent-child relationships and prosocial behaviors. Specifically, labor participation and a sense of social responsibility were identified as serial mediators in the relationship between parent-child relationships and prosocial behaviors among Chinese children. The results of this study provide more evidence in favor of the ecosystem theory and offer insights into the pathways linking family relationships to children’s mental well-being. To support Chinese children’s prosocial behaviors, intervention programs that strengthen parent-child relationships, labor participation, and a sense of responsibility should be combined.

Firstly, in support of H1a and H1b, the parent-child relationship demonstrated a positive association with prosocial behavior in Chinese children, which aligns with prior research on toddlers (Waugh et al., 2015) and middle childhood (Li et al., 2024). In early childhood, family psychosocial environmental factors such as the parent-child relationship are the core environmental factors influencing the formation and development of children’s prosocial behaviors (Ellis et al., 2011). Parents initially assume the role of external regulators in guiding the activity rhythms and emotions of early children. Over time, they gradually transition toward fostering their internal regulation, which serves as the foundation for the development of their socio-emotional competence (Liew, 2012). Children form a stable and secure attachment when parents respond to their needs in a warm and encouraging way. Children with strong attachment patterns are able to focus on the needs of others, develop empathy for them, and extend assistance and support (Shaver and Mikulincer, 2005). It means that the establishment of positive parent-child relationships creates a stable foundation for children to actively participate in social interactions and display prosocial behaviors. Furthermore, according to Elhusseini et al. (2023) children raised in an environment characterized by a significant level of harmony have the capacity to cultivate positive emotional experiences. These experiences facilitate individuals in deriving enjoyment from their social interactions and foster a more accurate and optimistic perception of their surroundings and environment, which means they are more likely to exhibit prosocial behaviors.

Second, our results suggest that labor participation mediates the association between the parent-child relationship and children’s prosocial behavior, supporting H2a and H2b. Research indicates that a variety of factors influence children’s willingness to help around the house, with parental encouragement being the primary factor (Antill et al., 1996). A strong parent-child relationship is essential for this kind of encouragement to be effective, which means labor participation inside the family and parent-child relationships are strongly correlated. Children’s labor participation is the process of interaction with the environment and others (White and Mistry, 2016). Labor participation, particularly in household chores, offers children the chance to engage in work and collaborate with others (Li S., 2016). When children feel that their opinions are respected in the process of participation, it is conducive to the gradual cultivation of children’s sense of responsibility and has a further positive impact on children’s participatory behaviors in community governance and building (Blair, 1992). That is to say, when labor participation is used as an educational tool to promote children’s development, by fostering common goals and cultivating a collaborative atmosphere, children derive satisfaction from engaging in cooperation and assisting others. They understand the value of helping others, which promotes their prosocial behavior (Grusec et al., 1996).

Furthermore, the findings indicate that social responsibility acts as a mediator between the parent-child relationship and prosocial behavior, providing support for hypotheses H3a and H3b. This finding further supports Eisenberg’s prosocial moral theory (Eisenberg, 2014). From a theoretical perspective, warm and secure parent-child relationships provide a foundation for children to develop empathy and internalize moral values. Through responsive interactions, parents model prosocial norms and create opportunities for perspective-taking, facilitating children’s progression from external compliance to internalized social responsibility. Previous studies have also shown the beneficial impact of moral feeling on fostering individual behavior (Thomson and Siegel, 2013). Specifically, cultivating children’s consciousness of social responsibility not only encourages their active engagement and inclusion in groups but also greatly enhances the demonstration of prosocial behavior (Du et al., 2024). The development of children’s sense of responsibility is shaped by various factors, with the parent-child relationship being particularly influential. This relationship serves as the foundation for children to establish emotional bonds with others, and it plays a crucial role in fostering their moral emotions and promoting their social responsibility (Maiya et al., 2023). Furthermore, the influence of social responsibility as a mediator accounted for 39.07% and 51.45% of the total effect, respectively. This suggests that enhancing the emotional bond among family members has a beneficial impact on fostering children’s social responsibility and facilitating their socialization. These several variables collaborate to facilitate the development of youngsters into responsible persons who are capable of making valuable contributions to society.

Finally, labor participation and sense of social responsibility have a chain mediating effect between father-child/mother-child relationships and prosocial behavior, supporting hypotheses H4a and H4b. Increased labor participation was positively associated with an increased sense of social responsibility, consistent with the results of existing studies (Hossain et al., 2023). Labor participation, by assuming and performing basic responsibilities, enables children and adolescents to experience the importance of contributing to society, thus promoting the formation and development of social responsibility awareness. What’s more, one of the most interesting findings of our current study was that, unlike father-child relationships, mother-child relationships and children’s prosocial behavior are mediated by labor participation and social responsibility after controlling for mediating variables. Consistent with our findings, previous research has shown that father-child relationships tend to directly influence children’s behavioral patterns (Ferreira et al., 2016). Particularly, frequent father-child interactions during middle childhood may play a significant role in shaping children’s emotional and physiological regulatory processes like stress reactivity and emotion regulation, which are critical for the effective management of behavioral problems (Kamza and Putko, 2021). However, the mother-child relationships may have a more foundational and pervasive influence through daily caregiving and emotional socialization on children’s social-emotional functioning, (Ferreira et al., 2016) with mothers more likely to promote children’s academic and social-emotional skills development than fathers (Howard et al., 2006). Given that parents serve as the primary social influencers for children, particularly in terms of moral development and prosocial behavior, cultivating a healthy parent-child relationship becomes crucial. A healthy parent-child relationship can promote children’s labor participation, nurture adolescents’ sense of social responsibility, and ultimately foster prosocial behavior. Therefore, parents should pay more attention to the cultivation of labor participation and social responsibility when cultivating and educating their children’s prosocial behavior.

Collectively, the present research indicates that father-child/mother-child relationships, labor participation, and sense of social responsibility are interconnected in the formation of prosocial behavior in children. The findings of this study suggest that a positive parent-child relationship is positively associated with children’s prosocial behavior. Additionally, the study uncovers the underlying psychological mechanism through which the parent-child relationship influences prosocial behavior. These results serve as a foundation for enhancing the cultivation of prosocial tendencies and promoting mental health education among children and adolescents.

## Limitations

The current study has presented new ideas for improving prosocial behavior in children. However, it is crucial to recognize the limitations of this study. First, this current study is a cross-sectional survey, which means that it is limited in its ability to establish causal relationships between variables. To explore the relationship, future research can employ longitudinal tracking designs to examine the observed variable associations in this study. Second, the data for this study were obtained solely through self-reporting by participants, a method that is inherently subjective. As a result, it is possible that the relationships between variables may have been exaggerated. Third, the determinants associated with prosocial behavior exhibit a high level of intricacy. This study solely examines the labor participation and social responsibility mediators, and does not comprehensively elucidate the mechanisms underlying the association between the parent-child relationship and prosocial behavior in primary school pupils. Therefore, future research on the effects of other mediating variables is necessary. Fourth, both labor participation and prosocial behavior can be opportunity-dependent. Although our measures cover multiple contexts and assess behavioral tendencies rather than single-context frequencies, the study did not directly control for variations in opportunity structures across schools or families. Future research could incorporate school- or community-level variables to account for these contextual differences. Finally, it should be acknowledged that data were collected from only two primary schools, located in eastern and western China, respectively. Further examination and research are required to determine the generalizability of the findings from this study to other regions in China, other cultural contexts where collectivistic values may be less prominent, and other developmental periods where the mechanisms may operate differently.

## Conclusion

This study employed a serial mediation model to examine the associations among parent-child relationships and prosocial behaviors in Chinese children. The findings of this study indicate that the prosocial behavior of Chinese children was significantly and positively associated with the parent-child relationship. Moreover, labor participation and a sense of social responsibility were identified as serial mediators in the relationship between parent-child relationships and prosocial behaviors among Chinese children. The results of this study provide more evidence in favor of the ecosystem theory and offer new treatments or support pathways for the enhancement of children’s mental well-being. To support Chinese children’s prosocial behaviors, intervention programs that strengthen parent-child relationships, labor participation, and a sense of responsibility should be combined.

## Data Availability

The raw data supporting the conclusions of this article will be made available by the authors, without undue reservation.
